# The virtue of temperance: a neurobiological perspective

**DOI:** 10.3389/fnhum.2026.1674301

**Published:** 2026-04-22

**Authors:** Julia Palacios, Lauren Gordon, Erick Messias

**Affiliations:** Department of Psychiatry and Behavioral Neuroscience, Saint Louis University School of Medicine, St. Louis, MO, United States

**Keywords:** emotional regulation, forgiveness, neurobiology, neuroscience, temperance, prudence, humility

## Abstract

**Introduction:**

The virtue of temperance is a unique psychological characteristic that has been long regarded as a positive quality throughout many cultures. While it is colloquially recognized and appreciated, the neural underpinnings of temperance remain largely unexplored. In order to better understand the positive influence of this virtue, it would be useful to explore the following components of temperance: forgiveness, humility, prudence and emotional self-regulation. Our review outlines a theorized model connecting these components to specific neuroanatomical locations and overall psychological effects to highlight the areas of the brain associated with temperance.

**Methods:**

In reviewing the literature regarding the four components of temperance, a neurobiological model of temperance is proposed. Each component was analyzed independently, combining findings from various neuroimaging studies deemed relevant to the virtue of temperance.

**Results:**

Our neurobiological model for temperance highlights the intricate balance required between various reasoning areas of the brain and emotional centres. The reasoning areas of the brain involved with temperance include the following: dorsolateral prefrontal cortex located within the middle frontal gyrus, ventral/medial prefrontal cortex, inferior parietal lobule, supramarginal gyrus, temporoparietal junction, medial parietal cortex, and reduced activity of the posterior cingulate cortex. The emotional centers involved in temperance include the orbitofrontal cortex, parahippocampal gyrus, fusiform gyrus, dorsal/ventral cingulate regions, PFC-hippocampal-amygdala circuitry, paralimbic network and autonomic processes including the vagus nerve, sympathetic response and neuroendocrine systems. Namely, the vmPFC and dlPFC display significant overlap in relevant neurobiology and together represent all 4 components of temperance.

**Discussion:**

This review aims to open a scientific dialog about the virtue of temperance by defining positive psychological qualities and neurobiological activity of temperance in the human mind. Viewing temperance through the lens of neuroplasticity, is it suggested that the practice of temperance may alleviate symptoms across numerous mental disorders and even aid in disease prevention. Future work should utilize neuroimaging techniques such as fMRI/MRI and molecular transmission studies to examine each component more precisely and define areas of interaction between components. In doing so, the neural underpinnings of temperance proposed in this model can be refined to outline a dynamic pathway with targets for clinical intervention.

## 1 Introduction

Temperance has been defined by Peterson and Seligman as “the virtue of control over excess” (2004). At times it is used synonymously with the term “moderation,” implying an ability to regulate personal cognition, emotion and behavior. Previous studies have investigated the neurobiology of wisdom in order to propose a speculative model (Meeks and Jeste, 2009; Jeste and Lee, 2019), inspiring this research. Four components of temperance have been identified in the psychological literature: forgiveness, humility, prudence, and self-regulation (Peterson and Seligman, 2004), which will be discussed further in the context of neuroanatomy and physiology. Some of these components, such as forgiveness and self-regulation have already been discussed in the current scientific literature. Forgiveness has been found to hold clinical relevance by protecting against mood disorders such as depression and anxiety (Schuttenberg et al., 2022) and aiding in relapse prevention for substance use disorder (Berg et al., 2024; Scherer et al., 2011). Self-regulation is perhaps an even better example of a component of temperance that holds clinical relevance, as mindfulness practices are increasingly being incorporated in a therapeutic setting to aid in nervous system regulation. In this article, humility and prudence will be discussed similarly as potential therapeutic resources. The majority of studies reviewed in this paper have been conducted with task assignments and concurrent fMRI/MRI findings, making it possible to outline a framework of neurobiological regions associated with each trait.

Overall, this article aims to discuss each component of temperance, both in terms of clinical relevance and neuroanatomical findings, to reveal how the virtue of temperance could be utilized as a therapeutic target for individuals diagnosed with mood disorders and addictive disorders. In doing so, a model of associated neurobiology will be suggested to provide a framework for understanding its neural underpinnings and to inform further research and hypothesis testing.

## 2 Methodology

The idea for this article was conceived after reading Peterson and Seligman’s psychological perspective of temperance in *Character Strengths and Virtues* (2004). Temperance, now defined in scientifically-recognizable components, can be viewed from a neurobiological basis by conducting a review of neuroimaging studies. Considering that this article is a pioneering work in the field, the authors utilized a narrative approach to identify relevant literature with a wide scope. PubMed and Google Scholar databases were used to conduct searches using keywords such as “neurobiology,” “neuroimaging,” and “fMRI” in conjunction with each of the components of temperance. Included studies were cohort studies, observational studies, randomized clinical trials and literature reviews with pertinence to the primary research question. The majority of included studies focused on the adult population of human subjects, however some exceptions were made to include child and adolescent studies as well as animal research for topics that were unstudied in adult humans. Exclusion criteria included conference abstracts, editorials and studies deemed irrelevant to the research hypothesis. The included studies were then analyzed thematically and findings were synthesized to illuminate the theorized framework of temperance.

## 3 Neurobiology of the components of temperance

### 3.1 Forgiveness and mercy

Forgiveness is defined as an act of pardoning a fault, debt or wrongdoing and moving past a transgression (Hughes, 2015). It is regarded as a fulfilling act that is socially perceived as moral and beneficial to both the individual who forgives and the transgressor (Peterson and Seligman, 2004). Since the act of forgiveness requires one to choose goodwill instead of retaliation or avoidance, is it evident that the virtue of temperance plays a role in this process. When discussing the concept of forgiveness, it is important to differentiate between decisional and emotional forgiveness. In regard to decisional forgiveness, a meta-analysis by Fourie states that forgiving behavior among adults is associated with greater volume and functionality of the middle frontal gyrus (MFG), which includes the dorsolateral prefrontal cortex (dlPFC) (2020). This meta-analysis reports a correlation between the magnitude of the perceived offense and the amount of measured MFG activity, suggesting that the dlPFC directly modulates the decision to forgive a perceived external offense. For example, forgiving a grievance of high perceived magnitude was associated with more activity in this region of the brain when compared to forgiving an offense perceived as small or inconsequential (Fourie et al., 2020). The dlPFC is also associated with emotional regulation, executive functioning, working memory and selective attention (Sturm et al., 2016), suggesting potential involvement in both decisional and emotional forgiveness. To support the theorized involvement of the MFG in forgiveness, a recent fMRI study found that decisional forgiveness was positively associated with connectivity between the MFG and the left inferior parietal lobule (IPL) (Li et al., 2024). In comparison, this same study found emotional forgiveness to be associated with connectivity between the left supramarginal gyrus (SMG) and the right IPL.

These findings are further supported by a cross-sectional study outlining the relationship between MFG volume and mood disorders among adolescents, suggesting clinical relevance of forgiveness (Schuttenberg et al., 2022). In addition to finding a significant correlation between forgiveness and increased MFG volume, this study found that forgiveness plays a protective role against symptoms of depression and anxiety. These findings are relevant to help understand an age group that has an increased prevalence of mood disorders such as depression, anxiety and behavioral disorders when compared to the adult population (Ghandour et al., 2019). Another study found larger MFG volume to be associated with better cognitive control among adolescents (Burt et al., 2016). While one study has found an association between MFG volume and executive function among adults with dementia, there is not currently a study among healthy adult participants that clearly identifies a connection between forgiveness and MFG volume rather than activity (Krueger et al., 2011). Given the combined information above, the extrapolation can be made that the virtue of temperance is associated with greater MFG functioning and increased connectivity with the left IPL through the practice of decisional forgiveness, and increased connectivity between the right IPL and the left SMG through emotional forgiveness.

Associated Neurobiology: MFG of the dlPFC, bilateral IPL and left SMG.

### 3.2 Humility and modesty

Humility is defined by Peterson and Seligman as a “person’s own sense that he or she is not the center of the universe” (2004). While largely considered to be synonymous with modesty, there is slight nuance between these terms, with modesty being regarded as an external quality evidenced by behavior, and humility being an internal quality (Peterson and Seligman, 2004). For the sake of simplicity, this review will refer only to humility, with a focus on internal processes and behavioral components that contribute to temperance. Humility plays a role in temperance by informing personal and social behavior. While the neurobiology of humility has not been extensively studied, a recent conceptual article about relational humility suggests a multilayered definition of humility, including two subcategories that overlap with qualities of temperance: self-monitoring as a subsection of cognitive interpersonal humility, and limiting wants and desires as a function of ecological/interspecies humility (Narvaez, 2019). Self-monitoring plays important roles in both intrapersonal and interpersonal humility. Intrapersonal humility describes one’s ability to accurately assess their own achievements and worth, whereas interpersonal humility describes one’s ability to be appropriately attuned with another (Narvaez, 2019). Self-monitoring and limiting wants and desires have also been studied within the context of Reinforcement Sensitivity Theory (RST), specifically in regard to the behavior inhibition system (BIS) which aids in a cost–benefit analysis of conflict by monitoring internal goals, detecting threats or conflict cues and integrating these internal findings to support effective behavioral control (Kennis et al., 2013). The BIS largely relies on septo-hippocampal involvement within a PFC-hippocampal-amygdala circuit that guides output of behavior under conflict (McNaughton, 2025; Kennis et al., 2013). Specifically, hippocampal cells are thought to be directly activated by memories about available goals, which then receive environmental information and only release functional output when conflicts are identified between goals (McNaughton and Bannerman, 2024). The hippocampus is only involved if such a conflict exists, suggesting its role as a mediator of behavior inhibition. While this link has not been explored in the context of humility specifically, it suggests neurobiology of potential significance for self-monitoring and limiting wants and desires which could play an important role on a personal and social level by informing behavioral changes that are perceived positively by a group.

Another aspect important for maintaining intrapersonal humility is self-confidence, as one’s self-confidence equates to the understanding of his or her abilities and potential for improvement. Many studies that aimed to localize the neurobiology of self-confidence found increased activity in the orbitofrontal cortex (OFC) of rodents (Kepecs et al., 2008; Ott et al., 2018). These findings were further explored by Lak et al. (2014), who found that inhibition of the OFC in rats impaired metacognition, but had no effect on performance. This suggests that the OFC is associated with confidence about one’s decision, not the decision itself. Beer et al. (2010) further evaluated the role of the OFC in human participants, where they found that activity of the OFC associates with appropriate confidence in their reasoning in a forced-choice problem. These findings suggest that the role of the OFC in accurate self-confidence appears to be a neural basis for intrapersonal humility.

Intrapersonal humility contributes greatly to interpersonal humility, as humility within an interpersonal relationship requires a level of self-contentedness so each person has the capacity to focus on the other (Narvaez, 2019). In order to maintain interpersonal humility, it is important to harbor a strong self-other distinction. The self-other distinction is the ability to distinguish one’s own physical and mental states from another. This self-other distinction encompasses the ability to modulate how much one experiences the thoughts and emotions expressed by another. Van Overwalle (2009) proposed that the cortical region responsible for self-other distinction is the temporoparietal junction (TPJ), which consists of the superior temporal and inferior parietal cortex, with many researchers supporting this claim (Quesque and Brass, 2019; Steinbeis, 2016; Frewen et al., 2020). Quesque and Brass (2019) found that the TPJ and the medial prefrontal cortex (mPFC) play predominant roles in self-other distinction as well. In a 2013 fMRI study, self-referencing was significantly associated with mPFC activity for both positive and negative stimuli, further implying the mPFC in accurate self-assessment, lending to self-other distinction and social humility (Frewen et al.). Finally, Steinbeis (2016) extends these findings in his fMRI studies, finding that the posterior TPJ specifically functions to overcome cognitive egocentricity, whereas the anterior TPJ functions to overcome emotional egocentricity. It is also proposed that interpersonal humility is reliant on well-functioning neurobiology and self-regulatory systems, including the vagus nerve, stress response, and neuroendocrine systems (Carter and Porges, 2012). This area of research promises much room for growth, as the neurobiological pathways of humility have not been studied in an experimental setting or observed using brain imaging techniques.

Associated Neurobiology: OFC, TPJ, mPFC, PFC-hippocampal-amygdala circuit, vagus nerve, stress response, neuroendocrine systems.

### 3.3 Prudence

As a component of temperance, prudence provides one with the capacity to use future-oriented reasoning to control impulses and behave in a conscientious manner (Peterson and Seligman, 2004). Prudence can be thought of as an applied wisdom that is fueled by balanced thinking and conscientiousness rather than neuroticism (Peterson and Seligman, 2004). While the neurobiology of prudence has not been specifically explored in research, various components of prudence have been studied, including the following: conscientiousness, goal-oriented cognition, and impulse control. One study found that individuals who scored highly in conscientiousness using the Big Five personality scale (Costa and McCrae, 1997) had statistically significant increases in cortical thickness in the following regions: bilateral parahippocampal gyrus, bilateral fusiform gyrus (FG), left dorsal cingulate gyrus, right ventral anterior cingulate cortex (aCC), right medial OFC, and left dorsomedial prefrontal cortex (dmPFC) (Lewis et al., 2018). A study on individuals with traumatic brain injuries revealed that damage to the left dlPFC was associated with high neuroticism and low conscientiousness (Forbes et al., 2014). However, these areas of the brain cannot be clearly tied to prudence, as increased activity in the dlPFC and right hippocampal gyrus has also been associated with increased impulsive behavior and cravings in pathological gambling subjects (Crockford et al., 2005; Williams and Potenza, 2008), which one would not expect to see among prudent individuals who tend to resist instant gratification. It is evident that both under-activity and over-activity of the dlPFC are tied to pathological thought and behavior, suggesting that this area of the brain requires some degree of modulation and interaction with other regions of the brain to produce prudent behavior. This idea is supported by a study from Zhejaian University that outlined two distinct circuits between the dlPFC and OFC that process risk and ambiguity, respectively, in the context of decision-making (Yang et al., 2017).

When considering conscientiousness in the context of prudence, it is also important to discuss the interaction between neuroticism and conscientiousness, as a form of “healthy neuroticism” has been theorized in the current literature. While Peterson and Seligman note that prudent individuals tend to display lower than average neuroticism (2004), a recent meta-analysis draws together data suggesting that individuals with high neuroticism and high conscientiousness tend to practice an increased number of health behaviors and experience more positive health outcomes (Graham et al., 2020). A 2016 study examined neuroticism in the context of prudence, presenting study participants with binary choices between risky lotteries and measuring emotional arousal via FaceReader software (Breaban et al., 2016). Ultimately, among their sample size of eighty-three students, they found no significant correlation between prudence and neuroticism. However, they did draw significant positive correlations between prudence and emotional arousal, with more intense emotions such as surprise and disgust correlating with greater prudence. This suggests that prudent behavior relies on an interplay between emotional arousal and perhaps “healthy neuroticism” to some degree, although this association could benefit from being studied among larger sample sizes.

Goal-oriented behavior, another component of prudence, is theorized to require PFC modulation of subcortical systems to respond appropriately to situations with emotional or contextual interference (Hare and Casey, 2005). This study also proposes increased communication between the PFC and amygdala (Am) to avoid negative consequences and increased interaction between the PFC and ventral striatum (VS) to enhance the selection of positive outcomes or rewards. The final aspect of prudence to discuss is impulse control. As cited earlier, the aCC may be involved in conscientiousness as a function of prudence (Lewis et al., 2018), and additionally, a 2015 fMRI study highlights the importance of the aCC in impulse control, another key aspect of prudence (Sohrabi et al.). This study demonstrated that increased activity of the aCC was found to enable greater control of risk-taking behaviors and improved contextual awareness of chance relative to gambling tasks.

Goal-oriented behavior and impulse control are also discussed within Reinforcement Sensitivity Theory (RST) with the BIS implying activity of the septo-hippocampal system in regulating avoidant behavior within the context of conflicting goals (Reuter et al., 2015; Gray, 1982). This differs from the findings of Hare and Casey (2005) which imply “top down” modulation of subcortical systems by the PFC and instead suggests hippocampal regulation of interaction between the PFC and amygdala. In essence, if a certain activity is deemed incompatible with a prudent goal, the hippocampus will aid in controlling impulsive behavior that would conflict with the desired outcome. Both theories lend insight to how emotional stimuli influence behavior. Whether prudent behavior occurs through a “top-down” or “bottom up” method likely depends on multiple factors, including emotional reactivity, one’s perception of prudence, whether or not one’s behavior is motivated by a prudent end goal and whether this goal exists in the presence of conflicting goals. Like all components of temperance, these variables do not have a standardized definition within the current literature. Consequently, the methods for measuring prudence have limitations and often rely on scaling systems such as the Five-Factor Model of personality, which has limited external validity. Further research utilizing fMRI and other imaging modalities to study tasks associated with subcomponents of prudence, such as conscientiousness and goal-oriented behavior, would likely provide a better foundation for understanding which brain regions directly support prudence. Additionally, those who practice increased health behaviors compared to others could potentially be studied retrospectively to identify regions of the brain with increased volume or activity compared to controls.

Associated Neurobiology: FG, left dorsal cingulate gyrus, right ventral aCC, right medial OFC, PFC, Am, VS, right hippocampal gyrus, septo-hippocampal system, dlPFC.

### 3.4 Self-regulation and emotional homeostasis

Emotional self-regulation is a rapidly growing area of research recognized for its clinical relevance in the discussion of mental health. Emotional regulation through the practice of mindfulness-based interventions has been found to mitigate negative emotions and stress and contribute to effective coping strategies (Hofmann and Asmundson, 2008). Mindfulness-based practices inherently involve self-awareness, a necessary aspect of self-regulation since a basic awareness of one’s emotional landscape is required to seek homeostasis. A review by Lou et al. (2017) proposes the underlying cortical centers involved in self-awareness. They found that the medial paralimbic network may be the common neural circuitry for self-awareness, specifically outlining two “hubs.” These hubs include the PFC, and aCC along with the medial parietal cingulate (mPC) and posterior cingulate cortex (pCC). These regions have a high metabolic activity at rest and decreased activity when one performs a goal-directed action in the external world, ultimately decreasing one’s self-awareness (Lou et al., 2017). A 2019 review of fMRI neurofeedback studies further supports this data, with the aCC being active in relation to various emotional regulation tasks (Linhartová et al.). In a neurofeedback study, the PFC and parahippocampal gyrus are once again outlined as relevant neurobiology, as patients are tasked with recalling positive memories to self-regulate (Zhu et al., 2019). These findings suggest that an intricate interaction between higher-order centres of the brain (PFC, mPC, pCC, aCC) and paralimbic structures play a major role in self-awareness, and ultimately self-regulation.

While multiple models of emotional self-regulation have been proposed over the years, evidence of a dual-process model has emerged through neuroimaging studies which supports the idea of a “top-down” modulation of cortical regions in congruence with “bottom-up” control of emotional reactivity (Ochsner and Gross, 2005). More specifically, it is proposed that activation of the ventral PFC and OFC evaluate stimuli to determine an appropriate response, whereas the dorsal PFC functions as an emotional appraisal system. Within the context of practicing temperance, it would be imperative to properly evaluate emotional stimuli to produce an appropriate response, supporting the relevance of these findings in relation to this review. Additionally, a meta-analysis of thirteen fMRI studies found decreased activity of the pCC in association with acceptance as a feature of emotional homeostasis (Messina et al., 2021).

Associated Neurobiology: medial paralimbic network, PFC, aCC, pCC, mPC, OFC.

## 4 Results

Each component of temperance has been reviewed independently with proposed neurobiology identified. Forgiveness and cognitive control are both associated with increased activity in the MFG of the dlPFC and possible increased volume of the MFG. There is also increased connectivity between the bilateral IPL and MFG and left SMG for decisional and emotional forgiveness, respectively. Further, our results define areas of the brain associated with humility, which is likely the least studied component of temperance. Humility likely relies on the OFC (intrapersonal) and may be supported by activity in the TPJ and mPFC (interpersonal). Interpersonal humility also involves activity by the vagus nerve, stress response and neuroendocrine systems, although this relationship has not been well defined. Prudence is associated with many areas of the brain, including increased activity in the FG, left dorsal cingulate gyrus, right ventral aCC, right medial OFC, right parahippocampal gyrus and dlPFC, with a balance of dlPFC activity being required to appropriately affect impulse control and overall conscientiousness. Communication between the PFC, amygdala and ventral striatum is likely increased to promote balanced decision making in prudence as well. Emotional self-regulation is associated with modulation of self-awareness through activity of the medial paralimbic network, involving the PFC, aCC and pCC along with the medial parietal cortex. Emotional regulation is also associated with activation of the ventral PFC, OFC (emotional response), dorsal PFC (stimulus appraisal) and decreased activity of the pCC (emotional homeostasis).

Notably, in reviewing these findings, some areas of the brain were associated with two or more components of temperance, especially between prudence and the other components. For example, the vmPFC is associated with decisional prudence, emotional regulation and interpersonal humility. The dlPFC is associated with forgiving behavior, prudent conscientiousness, and stimulus appraisal in emotional regulation. Other regions represented at least two components. These include the dmPFC (self-regulation, interpersonal humility), mOFC (prudence, intrapersonal humility), aCC/parahippocampal gyrus (prudence, self-regulation), PFC-hippocampal-amygdala circuit (humility, prudence) and the VS (interpersonal humility, prudence). Overall, these results demonstrate a strong role of the PFC in temperate thought and behavior in addition to the importance of prudence as a component within the context of temperance.

## 5 Discussion

### 5.1 Clinical and therapeutic relevance of temperance

**Figure 1:**
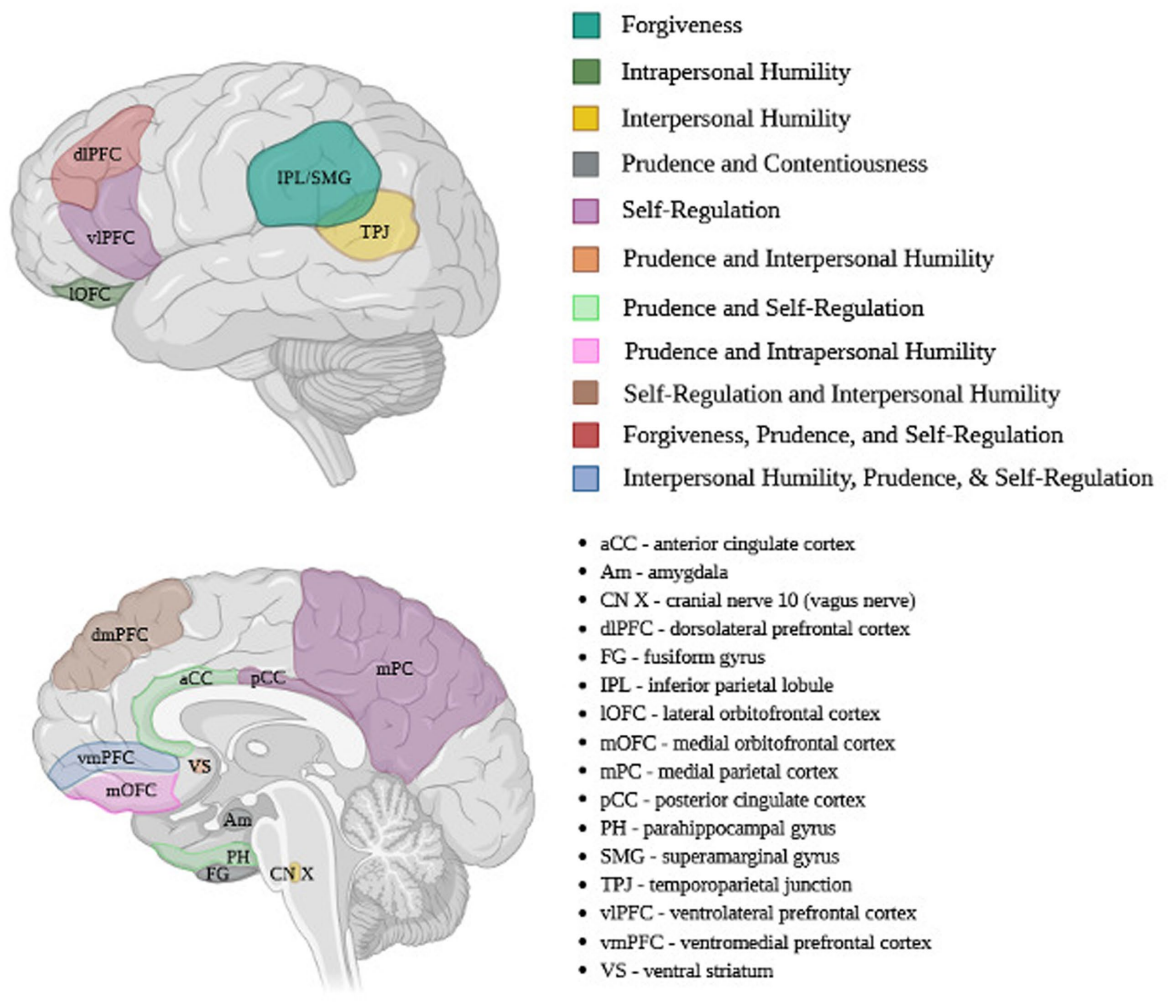
Neuroanatomical model for temperance. Created in BioRender. Gordon (2015) https://BioRender.com/fyofzjj

This review proposes a novel neuroanatomical model for the virtue of temperance by synthesizing current findings on its components (Table 1; Figure 1). Although still in its early stages, this neuroanatomic framework for temperance and its components provides an illustration of the neuromodulatory role of temperance on the brain. Similar to muscle hypertrophy, neurons strengthen their connections between brain regions with repeated stimulation, a concept known as “neuroplasticity” (Fuchs and Flügge, 2014; Mateos-Aparicio and Rodriquez-Moreno, 2019). This concept was first introduced by Satiago Ramon y Cajal in the early 1900s which was later expanded and popularized by Donal Hebb in 1949. Strengthening neuronal connections by practicing a virtue promises many potential benefits, as demonstrated by gratitude practice for mitigating depressive symptoms and increasing subjective happiness and life satisfaction (Cunha et al., 2019). Applying this principle to the virtue of temperance, it can be theorized that the more one practices temperance, either through forgiveness, humility, prudence, or emotional regulation, the less effort required to engage in this pattern in the future. The practice of temperance poses great potential for alleviating various mental disorders, as many conditions demonstrate decreased temperance in at least one facet. This does not equate to a lack of virtue in individuals suffering from these disorders but instead demonstrates a lack of tools to help cope with and overcome some symptoms associated with certain diagnoses. Additionally, utilizing a positive psychology trait for therapeutic benefit provides an affordable and widely accessible avenue for developing these tools and facilitating healing.

**Table 1:** Theorized model of neurobiological activity for the virtue of temperance.

Components of temperance	Trait characteristics	Neuroanatomical location or physiological pathway
Forgiveness	Behavior, cognitive control	Middle frontal gyrus of the dorsolateral prefrontal cortex
	Decisional	Middle frontal gyrus and left inferior parietal lobule
	Emotional	Right inferior parietal lobule and left supramarginal gyrus
Humility	Intrapersonal (self-confidence, self-monitoring)	Orbitofrontal cortex, septo-hippocampal system
	Interpersonal (self-other distinction, limiting wants and desires)	Temporoparietal junction, medial prefrontal cortex; vagus nerve, sympathetic response, neuroendocrine systems, PFC-hippocampal-amygdala circuit
Prudence	Conscientiousness	Parahippocampal gyrus, fusiform gyrus, left dorsal cingulate gyrus, right ventral anterior cingulate cortex, right medial orbitofrontal cortex, left dorsomedial prefrontal cortex
	Goal-oriented behavior, decision making	Prefrontal cortex modulation of amygdala and ventral striatum, septo-hippocampal system
Emotional regulation	Self-awareness	Medial paralimbic network: Prefrontal cortex, anterior, and posterior cingulate cortex, medial parietal cortex
	Stimulus appraisal	Dorsal prefrontal cortex
	Emotional response	Ventral prefrontal cortex
	Emotional homeostasis	Reduced posterior cingulate cortex activity

Individual components of temperance correspond to areas of activity proposed by in-depth review of scientific literature.

Temperance largely equates to moderation in one’s thoughts and actions. Many mental disorders demonstrate an imbalance of control, either through one’s perceptions, thoughts, emotions, or behaviors. For example, individuals with anxiety disorders and specific phobias demonstrate limited control over their perception of real or anticipated stimuli and subsequent emotional responses (American Psychiatric Association, 2022). Substance-related disorders demonstrate limited control over desires and behaviors due to intense activation of the reward pathways which may impair brain inhibitory mechanisms (American Psychiatric Association, 2022). Obsessive-compulsive disorders demonstrate limited control over thoughts and the need to carry out ego-dystonic actions to alleviate those thoughts (American Psychiatric Association, 2022). Many of these disorders are treated with medical interventions, as many mental disorders require medications for appropriate symptom management. However, many patients receive cognitive behavioral therapy (CBT) either independently or in combination with pharmacotherapy to gain tools for managing a wide array of conditions ranging from depression to substance use disorders (Gautam et al., 2020; Magill et al., 2023). Practicing temperance, especially in a guided CBT format, could potentially provide patients with additional tools necessary to manage their symptoms and build control to overcome maladaptive habits.

One mental disorder we have begun to recognize may benefit from training temperance includes substance use disorders. Research recognizes that a wide variety of substances can alter the neurobiological pathways described in this paper when used over time. This is demonstrated both when people try to resist cravings, and thus practice temperance, or engage in substance use. For example, studies show that individuals who actively regulate their substance cravings demonstrate increased activation of the aCC, dlPFC, vmPFC, and the vlPFC (Tabibnia et al., 2011; Wilcox et al., 2016). Each of these regions correspond to at least one component of temperance described in the proposed model. Additionally, engaging in substance use alters this activation and connectivity, where individuals with substance use disorders often have decreased functional connectivity between the nucleus accumbens (a key part of the reward system) and many of the regions associated with cognitive-behavioral control, such as the dorsal aCC, dlPFC, and inferior FG (Motzkin et al., 2014). This decrease in connectivity suggests impaired behavioral inhibition, as the frontal cortices cannot properly connect with the reward system, including the nucleus accumbens. However, this modulation related to substance use is not permanent, as Tanabe et al. (2019) found that length of abstinence from substances positively correlates with connectivity to the dlPFC and negatively correlates with connectivity to subcortical regions. This equates to greater cognitive control and decreased emotional influence over time as one abstains from substance use. These findings serve as the foundation for exploring ways to practice temperance as a form of therapy for substance use disorders, which is already being done in some capacity.

For example, studies find that individuals with substance use disorders who engage in self-forgiveness therapy were better at preventing relapse after intervention (Berg et al., 2024; Scherer et al., 2011). Additionally, having individuals with substance use disorder practice self-efficacy (e.g., Kadden and Litt, 2011; Yang et al., 2019), a practice of intrapersonal humility, and volunteering (e.g., Rady et al., 2018), a practice of interpersonal humility demonstrated increased success with refraining from substance use. Although the examples described above were given in the form of coaching, there is potential for these therapies to be utilized in a procedural setting if relevant neurobiology can be identified and targeted by transcranial stimulation or deep brain stimulation. Further, these studies merely address substance use disorders, however real-time fMRI neurofeedback for enhancing emotional regulation has shown to significantly reduce symptoms for patients with depression, anxiety, PTSD, BPD and schizophrenia (Linhartová et al., 2019), lending to the idea that temperance could provide benefits for many different types of disorders. Emotional regulation strategies have also been targeted more narrowly in CBT to effectively manage depression (Forkmann et al., 2014) and in Dialectical Behavioral Therapy (DBT) to address emotional dysregulation associated with borderline personality disorder (Goodman et al., 2014). Additionally, prudence, another component of temperance, may have a role within disease prevention. Some studies have equated conscientiousness, a key component of prudence, with better health behaviors and outcomes when combined with healthy neuroticism (Graham et al., 2020). Generally speaking, individual components of temperance have already demonstrated promising effects for various mental health disorders when studied independently, but the combined effects of these components remain unknown and likely hold even more value when practiced concurrently. The more temperance as a whole can be understood from a neurobiological standpoint, the more it can be utilized in a controlled therapeutic environment. From symptom management to disease prevention, it is undeniable that temperance-targeting therapies suggest a host of clinical benefits.

### 5.2 Limitations

This is the first paper to begin outlining the neurobiological basis for temperance. In this review, we primarily focused on the neuroanatomical mappings of forgiveness, humility, prudence, and emotional regulation using previous MRI/fMRI and related studies mapping neural activation during task completion. As useful as these studies are, most studies do not explicitly measure many components of temperance, including humility and prudence. This required us to frame these components of temperance using previous research as a proxy, rather than deriving novel data to support the neurobiology of temperance. Further, our findings primarily focus on activation of brain regions during specific tasks related to temperance. This means our review exclusively studies association rather than causation since the data does not exist to definitively map neurotransmission between brain regions and temporal mechanisms of activation for temperance. While these aspects of our research limit its current clinical applicability, it is worth noting that they fall outside of its intended scope. The simple neurobiological framework proposed in this article is a crucial prerequisite for any future work in this area.

### 5.3 Direction for future research

On a broad scale, it is evident that this virtue requires an intricate balance between various reasoning and emotional centers of the brain. Namely, the vmPFC and dlPFC are associated with more than one component of temperance, offering a promising direction for future research. Additional research is required to complete this neurobiological framework of temperance. Specifically, subsequent studies should primarily focus on functional processes when completing specific tasks associated with forgiveness, humility, prudence, and emotional regulation as defined by Peterson and Seligman (2004). Many of the studies cited in this article have already utilized fMRI/MRI to understand the neurobiology of the components of temperance. For forgiveness, the following tasks have been utilized: judging blameworthiness versus forgivability, selecting more forgivable explanations for described situations, economic decision making, reading narratives of hurtful events followed by an indication to forgive or hold a grudge, choosing to forgive an opponent in a monetary game and choosing to forgive a mild interpersonal transgression (Fourie et al., 2020; Li et al., 2024). For humility, self-appraisal tasks have been utilized that involve viewing pictures of themselves or others and saying “I am” or “he/she is” adjectives, repeating introspective statements regarding personality, behavior, values (Frewen et al., 2013; Steinbeis, 2016). Prudence is perhaps as equally understudied as humility and thus has limited testing under fMRI tasking. However, Sohrabi et al. studied impulse control tasks involving risky gambling decisions among participants in 2015. The conscientiousness and goal orientation aspects of prudence remain largely unstudied. To study conscientiousness, participants could undergo fMRI while being instructed to think about performing a kind gesture for a stranger. To study goal orientation, structural MRI could be performed in individuals who have sought and achieved positive health outcomes to analyze the volume of the PFC, amygdala and ventral striatum circuitry associated with this aspect of prudence, which may also serve as a measure of conscientiousness and neuroticism as well. Additionally, subjects could be presented with a choice between two conflicting goals requiring a cost–benefit analysis in order to study self-monitoring and goal-oriented behavior within the context of humility and prudence. Fortunately, for emotional self-regulation, fMRI tasking has already been performed with participants retrieving positive personal memories to modulate emotional responses (Zhu et al., 2019) and reinterpreting meaning of images or scenarios to be less negative (Sohrabi et al., 2015).

Studying temperance as a composite of these components under fMRI/MRI has not yet been attempted, as it is nearly impossible to define a single task that could incorporate the entire psychological process of this virtue. However, the neurobiological model proposed in this study could be tested by having participants perform fMRI tasks outlined above for each component and its associated aspects under the umbrella of one study. Further, temperance could also be studied via prospective studies of individuals practicing behaviors that model multiple qualities of temperance in order to identify regions of the brain that increase in mass versus atrophy over time. One suggestion would be to study patients pursuing recovery from substance use disorders, since this requires an inherent need for humility to recognize maladaptive tendencies, self-forgiveness and prudence to choose positive, ego-syntonic habits and inhibit desires, and emotional regulation to prevent relapse, all key components of temperance. Due to the complicated nature of addiction and sobriety, it would likely be difficult to limit confounding variables in this type of study design, and for that reason, task-oriented fMRI studies of individual components of temperance would yield cleaner, more usable data to study the neurobiological model of temperance.

Additionally, molecular studies should be done to more specifically outline the neurotransmission involved in these processes of temperance. While neurotransmission has not been explicitly studied in conjunction with temperance or its components, our models suggest potential involvement of dopaminergic regulation to aid in reward processing and gamma-aminobutyric acid (GABA) signaling to hone behavioral inhibitory control. Both of these neurotransmitters are dysregulated in behavioral addictive disorders, implying that some degree of regulation is required to achieve temperate behavior (Peng et al., 2025). Yet, many other neurotransmitters are likely involved, with serotonin (5-hydroxytryptamine, 5-HT), glutamate and endorphins being additional candidates to investigate. Finally, additional research is required to better define how these components of temperance influence one another. The current model specifically looks at each component in isolation and yet they are likely interconnected. In this article, certain aspects of the components overlap with each other in concept and in neurobiology, such as emotional regulation being involved in the processes of forgiveness and self-regulation, which both display dlPFC activation. Additionally, humility likely lends to other components. For example, self-referencing is necessary for both interpersonal humility and emotional self-regulation, which display overlapping neurobiology involving the mPFC. Behavioral inhibition is likely a shared component between humility and prudence in terms of self-monitoring and behavioral inhibition. Our article has discovered significant overlap in the neurobiology of some components, especially the vmPFC and dlPFC which together represent all 4 components of temperance. Future studies should investigate how activation of brain regions associated with one component of temperance influences the activation of brain regions associated with a different component. This current neuroanatomical model of temperance provides a strong foundation for these future objectives and will provide direction in demonstrating how these findings can be applied in a clinical setting moving forward.

## 6 Conclusion

This review proposes a novel neuroanatomical model for the virtue of temperance by synthesizing current findings on its components (Table 1; Figure 1). On a broad scale, it is evident that this virtue requires an intricate balance between various reasoning and emotional centers of the brain. Namely, the dlPFC is associated with more than one component of temperance, offering a promising direction for future research. The benefits of understanding temperance are undeniable. One simply needs to look at the negative impact of conditions associated with a lack of impulse control such as substance abuse disorders. Once we can clearly define how a temperate brain functions, it may be possible to target these pathways in future therapeutic interventions. As with many virtuous traits, temperance can be difficult to clearly define and measure, emphasizing one limitation of this review. Moving forward, it would be beneficial to analyse cognitive or emotional tasks associated with temperance using fMRI paradigms or other brain imaging techniques to more directly outline dynamic pathways associated with temperance. It may even be possible to utilize neurostimulation to support some of these components associated with better mental health outcomes. With time, perhaps we will find the words of Genevan philosopher Rousseaue (1762) to ring true: “Temperance and labor are the two best physicians of man; labor sharpens the appetite, and temperance prevents from indulging to excess.”

## Data Availability

The original contributions presented in the study are included in the article/supplementary material, further inquiries can be directed to the corresponding author.

## Glossary

aCC: anterior cingulate cortex
Am: amygdala
BIS: behavioral inhibition system
CBT: cognitive behavioral therapy
CN X: cranial nerve 10 (vagus nerve)
DBT: dialectical behavioral therapy
dlPFC: dorsolateral prefrontal cortex
FG: fusiform gyrus
fMRI: functional magnetic resonance imaging
GABA: gamma-aminobutyric acid
IPL: inferior parietal lobule
lOFC: lateral orbitofrontal cortex
MFG: middle frontal gyrus
mOFC: middle orbitofrontal cortex
mPFC: medial prefrontal cortex
mPC: medial parietal cortex
MRI: magnetic resonance imaging
OFC: orbitofrontal cortex
pCC: posterior cingulate cortex
PFC: prefrontal cortex
PH: parahippocampal gyrus
RST: reinforcement sensitivity theory
SMG: supramarginal gyrus
TPJ: temporoparietal junction
vlPFC: ventrolateral prefrontal cortex
vmPFC: ventromedial prefrontal cortex
VS: ventral striatum
5-HT: 5-hydroxytryptamine
